# Model-driven exploration of poro-viscoelasticity in human brain tissue: be careful with the parameters!

**DOI:** 10.1098/rsfs.2024.0026

**Published:** 2024-12-06

**Authors:** Alexander Greiner, Nina Reiter, Jan Hinrichsen, Manuel P. Kainz, Gerhard Sommer, Gerhard A. Holzapfel, Paul Steinmann, Ester Comellas, Silvia Budday

**Affiliations:** ^1^ Department of Mechanical Engineering, Institute of Continuum Mechanics and Biomechanics, Friedrich-Alexander-Universität Erlangen-Nürnberg, Erlangen, Germany; ^2^ Institute of Biomechanics, Graz University of Technology, Graz, Austria; ^3^ Department of Structural Engineering, Norwegian University of Science and Technology (NTNU), Trondheim, Trøndelag, Norway; ^4^ Department of Mechanical Engineering, Institute of Applied Mechanics, Friedrich-Alexander-Universität Erlangen-Nürnberg, Erlangen, Germany; ^5^ Glasgow Computational Engineering Centre, School of Engineering, University of Glasgow, Glasgow, UK; ^6^ Serra Húnter Fellow, Department of Physics, Universitat Politècnica de Catalunya, Barcelona, Spain; ^7^ International Center for Numerical Methods in Engineering (CIMNE), Barcelona, Spain

**Keywords:** poro-viscoelasticity, human brain, inverse parameter identification, permeability, constitutive modelling, Theory of Porous Media

## Abstract

The brain is arguably the most complex human organ and modelling its mechanical behaviour has challenged researchers for decades. There is still a lack of understanding on how this multiphase tissue responds to mechanical loading and how material parameters can be reliably calibrated. While previous viscoelastic models with two relaxation times have successfully captured the response of brain tissue, the Theory of Porous Media provides a continuum mechanical framework to explore the underlying physical mechanisms, including interactions between solid matrix and free-flowing interstitial fluid. Following our previously published experimental testing protocol, here we perform finite element simulations of cyclic compression–tension loading and compression–relaxation experiments on human brain white and gray matter specimens. The solid volumetric stress proves to be a crucial factor for the overall biphasic tissue behaviour as it strongly interferes with porous effects controlled by the permeability. An inverse parameter identification reveals that poroelasticity alone is insufficient to capture the time-dependent material behaviour, but a poro-viscoelastic formulation captures the response of brain tissue well. We provide valuable insights into the individual contributions of viscous and porous effects. However, due to the strong coupling between porous, viscous, and volumetric effects, additional experiments are required to reliably determine all material parameters.

## 1. Introduction

Despite decades of research, the human brain still poses exciting challenges for researchers from various fields. More recently, there is increasing interest in the role of mechanical signals for brain development [1–3], injury [4–6] and disease [7–10]. Modelling based on the theory of nonlinear continuum mechanics proves a valuable tool to computationally test hypotheses that complement experimental findings [11], to understand processes in the brain under physiological and pathological conditions [12] and to assist diagnosis and treatment of neurological disorders through personalized predictions [13–15].

Depending on the application, mechanical models for human brain tissue need to cover a wide range of time and length scales. Its highly heterogeneous, region-dependent microstructure relates to viscoelastic effects [16] and cannot be neglected for predictions on the organ scale [17]. Viscoelastic models with two relaxation times have been successful in capturing the time-dependent mechanical response of brain tissue under various loading conditions [11,18]. However, free-flowing interstitial fluid occupies a large fraction of the brain volume and contributes to the biomechanical response of human brain tissue through poroelastic effects [19–21]. For some applications, e.g. drug delivery in the brain during cancer treatment, it is essential to model the porous properties of brain tissue explicitly [22,23].

Existing poroelastic models are tailored to particular applications, e.g. tissue fracture [24], decompressive craniotomy [25], tumour growth and treatment [13], hydrocephalus [26] or drug delivery [22]. Models that treat brain tissue as a biphasic poro-viscoelastic material either focus on a specific experimental setup [27] or incorporate important analytical simplifications [28–30]. To our knowledge, the model described by [31] and the formulation proposed by our group [32] are the only approaches to date with the potential to capture the wide range of characteristics observed in the response of brain tissue under different biomechanical loading scenarios by modelling the brain as a poro-viscoelastic material.

Our versatile poro-viscoelastic model provides the possibility to describe and explore the underlying physical mechanisms within a biphasic material during mechanical loading, but identifying the associated model parameters becomes increasingly difficult with increasing model complexity. Even for poroelastic models (without accounting for viscoelastic effects) and without the specific application to brain tissue, the meaning of—and the correlation between—the individual model parameters seems poorly understood. In particular, parameters that control the volumetric response of the solid constituent of the biphasic material are chosen and interpreted in various fashions. For example, the shear modulus and the first Lamé parameter are often obtained through direct conversion from the Young’s modulus and the Poisson’s ratio values of brain tissue extracted from literature [22,23,33]. Pierce *et al.* [34] account for Lamé’s first parameter as a stress-like material parameter, which in the case of isochoric deformation of the solid matrix degenerates to a non-physical (positive) penalty parameter used to enforce incompressibility. Lucci *et al.* [35] mention that volumetric moduli penalize volumetric changes in the solid skeleton and acknowledge that their estimation is difficult. Indeed, due to the strong interaction between several parameters within a poro-viscoelastic formulation, currently available experimental data is not yet comprehensive enough to find unique material parameters.

In this study, we first carefully assess the physical meaning of poroelastic parameters in a poro-viscoelastic model based on the Theory of Porous Media. Thereby, we specifically analyse the correlation between volumetric constraints on the solid constituent and the porous effects in the overall biphasic tissue. We further discuss the size-dependency of poroelastic relaxation behaviour in a fully nonlinear setting. We then provide the basis to identify the free model parameters based on an inverse approach using the finite element method and experimental data from cyclic and stress relaxation experiments on human brain tissue from two different regions: visual cortex (grey matter) and corona radiata (white matter). We find that poroelasticity alone can capture important mechanical characteristics of human brain tissue, but is not sufficient to capture the highly hysteretic material response. Finally, we provide an outlook towards a reliable quantification of poro-viscoelastic material parameter sets in the future.

## 2. Material and methods

We apply our nonlinear poro-viscoelastic model [32] based on the Theory of Porous Media [36] to two different brain regions, i.e. the corona radiata (white matter) and the visual cortex (grey matter).

### 2.1. Experimental data

**Figure 1.**
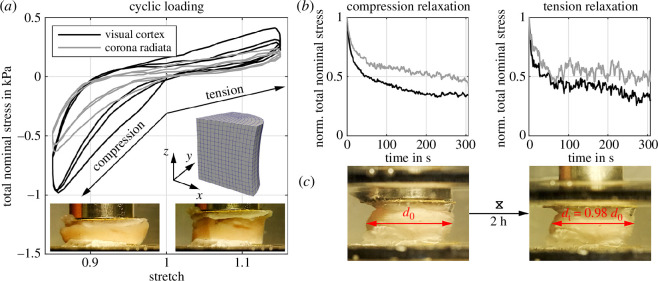
(*a*) Total nominal stress response during cyclic loading in compression and tension for samples extracted from the human visual cortex and corona radiata, respectively. In addition, we show a representative deformed specimen from the cortex and its corresponding finite element model. (*b*) Experimental results for compression relaxation and tension relaxation, normalized by the peak stress. (*c*) Lateral retraction of the deformation during long-term compression relaxation of 2 h for a corona radiata specimen. The final diameter $d_{t}=0.98\;d_{0}$ after relaxation is smaller than the initial diameter $d_{0}$ at the beginning of the relaxation period, measured with Fiji ImageJ [37].

As a reference, we use experimental data from large-strain compression and tension experiments performed on two cylindrical samples with a radius r=4mm, one extracted from the human visual cortex (grey matter) and one from the corona radiata (white matter), as illustrated in figure 1. The human brain tissue was extracted from a body donor (male, 71) who had given his written consent to donate his body to research and stored in artificial cerebrospinal fluid [38] until testing. The study was additionally approved by the Ethics Committee of Friedrich-Alexander-University Erlangen-Nürnberg, Germany, with the approval number 405_18 B. For details regarding the sample preparation and the experimental setup, we refer to [38]. We first applied three cycles of compression and tension with a loading velocity of $40\;\mu \;m\;s^{-1}$, and minimum and maximum overall vertical stretches of 0.85 and 1.15, corresponding to 15% strain in compression and tension, respectively (figure 1*a*). Subsequently, we performed a stress relaxation test, first in compression then in tension, at a maximum stretch of 0.85 and 1.15, respectively, with a loading velocity of $100\;\mu \;m\;s^{-1}$ and a holding period of 300s (figure 1*b*). We recorded the corresponding force in the direction of loading and determined the nominal stress as the force divided by the undeformed cross-sectional area of the specimen. The recorded data is subsequently preprocessed using a moving average filter and the Ramer–Douglas–Peucker algorithm [39], where the latter reduces the number of data points. To be able to perform tensile testing, we fully fixed the specimens to the upper and lower specimen holders using sandpaper and superglue. We tested the specimens fully submerged in phosphate buffered saline solution at 37°C. The pictures in figure 1*a* show a deformed specimen from the cortex at 15% compression (left) and 15% tension (right). Figure 1*c* demonstrates the lateral retraction of the deformation during long-term relaxation of 2 h for a corona radiata specimen extracted from a body donor (male, 57) who had given his written consent to donate his body to research. The final diameter $d_{t}=0.98\;d_{0}$ after relaxation is smaller than the initial diameter $d_{0}$ at the beginning of the relaxation period, measured with Fiji ImageJ [37].

### 2.2. Continuum kinematics

The biphasic brain tissue consists of a viscoelastic solid, representing the network of cells and blood vessels embedded within the extracellular matrix, fully saturated by free-flowing interstitial fluid. The individual solid and fluid constituents are assumed to be incompressible, while the overall compressibility of the biphasic material is captured by changing the solid and fluid volume fractions $n^{S}$ and $n^{F}$, respectively, subjected to the saturation condition $n^{S}+n^{F}=1$.

**Figure 2.**
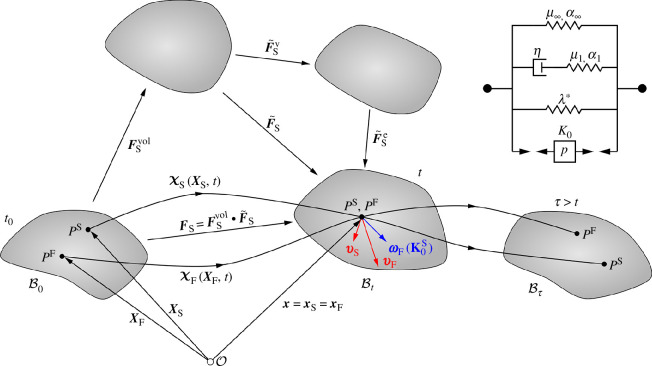
Kinematics of a biphasic material body within the context of the Theory of Porous Media [36]. Material particles of the solid and fluid components ($P^{S}$ and $P^{F}$, respectively) originate from different reference positions in the material configuration $B_{0}$ at initial time $t_{0}$, but occupy the same spatial position in the current configuration $B_{t}$ at time t. $B_{\tau }$ refers to the configuration at time τ>t. The deformation gradient ${𝑭}_{S}$ is multiplicatively split into volumetric ${𝑭}_{S}^{\mathrm{vol}}$ and isochoric ${\tilde{𝑭}}_{S}$ contributions. The isochoric part is decomposed into viscous ${\tilde{𝑭}}_{S}^{v}$ and elastic ${\tilde{𝑭}}_{S}^{e}$ parts. The seepage velocity ${𝒘}_{F}={𝒗}_{F}-{𝒗}_{S}=\partial {𝝌}_{F}/\partial t$−$\partial {𝝌}_{S}/\partial t$ describes the motion of the fluid with respect to the deforming solid. The rheological schematic indicates the solid-fluid interaction via a ‘porous' element depending on the pore pressure p and the initial intrinsic permeability $K_{0}$; $\mu_{\infty }$, $\mu_{1}$ and $\alpha_{\infty }$, $\alpha_{1}$ denote the Ogden shear moduli and nonlinearity parameters for the equilibrium and non-equilibrium part, respectively, while $\lambda^{*}$ is the first Lamé parameter and η the solid viscosity.


Following the Theory of Porous Media, the constituent deformation map reads $x=\chi_{S}(X_{S},t)=\chi_{F}(X_{F},t)$ and indicates that the material constituents originate from different reference positions $X_{S}$ and $X_{F}$ at time $t_{0}$, but occupy the same spatial position 𝒙 in the current configuration at time t (figure 2). We obtain the displacement of the solid component

(2.1) $$u_{S}=x-X_{S}$$

and its material deformation gradient


(2.2) $$F_{S}=\frac{\partial x}{\partial X_{S}}.$$

Importantly, the Jacobian of the solid deformation gradient $J_{S}=\det F_{S}>n_{0S}^{S}$ describes volumetric changes of the whole biphasic material, i.e. it also captures volumetric changes due to pore fluid flow. Once the Jacobian approaches the initial solid volume fraction $J_{S}⟶n_{0S}^{S}$, the point of compaction is reached, all pores are closed and the incompressibility constraint of the solid component prevents any further volume deformations [36].

### 2.3. Governing equations

We assume quasi-static loading conditions and neglect body forces and external tractions such that the weak form of the linear momentum balance in the reference configuration $B_{0}$ reads


(2.3) $$\int_{B_{0}}\nabla (\delta u_{S}):\tau \;dV_{0S}=0\;\;\forall \delta u_{S}.$$

The constitutive equation of the solid component renders the Kirchhoff stress tensor 𝝉, $\text{d}V_{0S}$ refers to the volume elements of the biphasic material in the reference configuration of the solid and $\delta {𝒖}_{S}$ are the solid displacement test functions. Since we do not prescribe fluid flow across the boundaries, the nonstationary, time-dependent mass balance equation reduces to


(2.4) $$\int_{B_{0}}\delta p{\dot{J}}_{S}\text{d}{\text{V}}_{0S}-\int_{B_{0}}\nabla (\delta p)\cdot w{\text{J}}_{S}\text{d}{\text{V}}_{0S}=0\;\forall \delta p\;.$$

The constitutive equation of the fluid provides the volume-weighted seepage velocity $𝒘=n^{F}{𝒘}_{F}$, ${\dot{J}}_{S}$ denotes the material time derivative of the Jacobian and δp are the pore pressure test functions.

### 2.4. Constitutive equations

We perform a multiplicative decomposition of the solid deformation gradient into elastic and viscous parts, i.e. ${𝑭}_{S}={𝑭}_{S}^{e}\cdot {𝑭}_{S}^{v}$ [40]. Similar to previous studies, we assume that the viscous contribution is purely isochoric, based on the premise that the volumetric response of brain tissue is primarily governed by fluid flow captured through poroelasticity. From a biophysical perspective, this modelling choice is motivated by the fact that the viscoelastic response is intended to solely represent the behaviour of the solid matrix, consisting of the extracellular matrix and the network of cells, inside which fluid is mostly trapped in the physiological state. We thus expect a purely isochoric viscous contribution, and the volumetric-isochoric decomposition in figure 2 is recovered through ${𝑭}_{S}={𝑭}_{S}^{e,vol}\cdot \tilde{{𝑭}_{S}^{e}}\cdot \tilde{{𝑭}_{S}^{v}}={𝑭}_{S}^{\mathrm{vol}}\cdot {\tilde{𝑭}}_{S}$. Then, the solid ‘extra’ stress ${𝝉}_{E}^{S}$ is split into (full) equilibrium (eq) and isochoric non-equilibrium (neq) parts and an additional volumetric (vol) contribution:


(2.5) $$\begin{array}{ccc} & 𝝉={𝝉}_{E}^{S}-pJ_{S}1={𝝉}_{E}^{\mathrm{eq}}+{𝝉}_{E}^{\mathrm{neq}}+{𝝉}_{E}^{\mathrm{vol}}-pJ_{S}1\;. & \end{array}$$

The fluid exerts a hydrostatic stress $\left(-pJ_{S}1\right)$ on the solid and 1 denotes the second-order unit tensor. Note that setting ${𝝉}_{E}^{\mathrm{neq}}=0$ in equation (2.5) reduces our nonlinear poro-viscoelastic model to a nonlinear poroelastic model. Based on previous studies [11,20,38], we choose a one-term Ogden model for the equilibrium and non-equilibrium parts. The equilibrium part of the Kirchhoff stress tensor is


(2.6) $$\begin{array}{ccc} & {𝝉}_{E}^{\mathrm{eq}}=\sum_{a=1}^{3}\beta_{\infty ,a}{𝒏}_{S,a}⊗{𝒏}_{S,a}\;\text{ with }\;\beta_{\infty ,a}=\mu_{\infty }\left[{\left(\lambda_{S,a}\right)}^{\alpha_{\infty }}-1\right]\;, & \end{array}$$

and depends on the principal stretches $\lambda_{S,a}$ and the eigenvectors ${𝒏}_{S,a}$ of the left Cauchy–Green strain tensor ${𝒃}_{S}={𝑭}_{S}\cdot {𝑭}_{S}^{⊤}=\sum_{a=1}^{3}\lambda_{S,a}^{2}{𝒏}_{S,a}⊗{𝒏}_{S,a}$. The constitutive parameters are the equilibrium Ogden shear modulus $\mu_{\infty }$ and the nonlinearity parameter $\alpha_{\infty }$. We formulate the non-equilibrium Kirchhoff stress tensor


(2.7) $$\begin{array}{ccc} & {𝝉}_{E}^{\mathrm{neq}}=\sum_{a=1}^{3}\beta_{1,a}{𝒏}_{S,a}^{e}⊗{𝒏}_{S,a}^{e}\;\text{ with }\;\beta_{1,a}=\mu_{1}\left[{\left({\tilde{\lambda }}_{S,a}^{e}\right)}^{\alpha_{1}}-\frac{1}{3}\left[{\left({\tilde{\lambda }}_{S,1}^{e}\right)}^{\alpha_{1}}+{\left({\tilde{\lambda }}_{S,2}^{e}\right)}^{\alpha_{1}}+{\left({\tilde{\lambda }}_{S,3}^{e}\right)}^{\alpha_{1}}\right]\right] & \end{array}$$

in terms of the isochoric elastic principal stretches ${\tilde{\lambda }}_{S,a}^{e}=[J_{S}^{e}{]}^{-1/3}\lambda_{S,a}^{e}$, the eigenvectors ${𝒏}_{S,a}^{e}$ of the elastic part of the left Cauchy–Green strain tensor ${𝒃}_{S}^{e}={𝑭}_{S}^{e}\cdot ({𝑭}_{S}^{e}{)}^{⊤}=\sum_{a=1}^{3}[\lambda_{S,a}^{e}{]}^{2}{𝒏}_{S,a}^{e}⊗{𝒏}_{S,a}^{e}$, the non-equilibrium Ogden shear modulus $\mu_{1}$ and the nonlinearity parameter $\alpha_{1}$. To ensure thermodynamical consistency, we assume isotropy and introduce an evolution equation


(2.8) $$\begin{array}{ccc} & -L_{{𝒗}_{S}}{𝒃}_{S}^{e}\cdot {\left[{𝒃}_{S}^{e}\right]}^{-1}=\frac{1}{\eta }{𝝉}_{E}^{\mathrm{neq}}\;, & \end{array}$$

where $L_{{𝒗}_{S}}$ denotes the Lie derivative along the velocity field of the solid motion and η is the solid viscosity, such that we *a priori* satisfy non-negative viscous dissipation power, i.e.


(2.9) $$D_{v}=\frac{1}{2\eta }\tau_{E}^{neq}:\tau_{E}^{neq}\geq 0\;for\;\eta >0.$$

The volumetric Kirchhoff stress contribution [36]


(2.10) $$\begin{array}{ccc} & {𝝉}_{E}^{\mathrm{vol}}=\lambda^{*}{\left[1-n_{0S}^{S}\right]}^{2}\left[\frac{J_{S}}{1-n_{0S}^{S}}-\frac{J_{S}}{J_{S}-n_{0S}^{S}}\right]1 & \end{array}$$

completes the definition of the solid stress tensor (2.5) and introduces the first Lamé parameter $\lambda^{*}$ of the solid component and the volume fraction of the solid component with respect to the solid reference configuration at the initial time, $n_{0S}^{S}$.

We compute the volume-weighted seepage velocity of the fluid with a Darcy-like law [41] according to


(2.11) $$\begin{array}{ccc} & 𝒘=-\frac{1}{\mu^{\mathrm{FR}}}\left[\frac{J_{S}-n_{0S}^{S}}{1-n_{0S}^{S}}\right]{𝑲}_{0}^{S}\cdot \nabla p, & \end{array}$$

where $\mu^{\mathrm{FR}}$ is the effective shear viscosity of the pore fluid and ${𝑲}_{0}^{S}=K_{0}1$ is the initial intrinsic permeability tensor, which we assume is isotropic. The porous dissipation power is


(2.12) $$D_{p}=\frac{\mu^{FR}}{K_{0}}\left[\frac{1-n_{0S}^{S}}{J_{S}-n_{0S}^{S}}\right]w\cdot w\geq 0\;,$$

and will always be non-negative, given that $\mu^{\mathrm{FR}}$ and $K_{0}$ are necessarily positive, $n_{0S}^{S}\in (0,1)$ and $J_{S}>n_{0S}^{S}$.

### 2.5. Numerical setup

The open source finite element library deal.ii [42] provides the numerical framework to reconstruct our large-strain cyclic loading and stress relaxation experiments introduced in §2.1. We discretize a quarter of our cylindrical specimens with 384 full integration Q2P1 elements, i.e. quadratic shape functions for the solid displacements, linear shape functions for the pore pressure and third-order Gaussian quadrature. We have performed a mesh refinement study to ensure that the simulation results were independent of the chosen mesh. The geometry dimensions are specimen specific for the visual cortex (r=4mm, h=3.4mm) and the corona radiata (r=4mm, h=5.0mm). The solid volume fraction is $n_{0S}^{S}=0.75$ and the fluid shear viscosity $\mu^{\mathrm{FR}}=0.89\;\mathrm{Pa}\cdot s$ [32,43]. The degrees of freedom at the bottom surface are fixed in space and a vertical displacement in the z-direction is applied to the top surface while being fixed in the x-y-plane (figure 1*a*). We apply symmetric boundary conditions to the flat lateral surfaces and only the cylinder hull is drained, i.e., fluid can only leave the sample through the cylinder hull and not through the spatially fixed (glued) top and bottom surfaces. Greiner *et al.* [43] describe the data analysis of the quantities (stresses, dissipation power, etc.) that we use to visualize and compare the finite element results.

In §3.2, we perform cyclic loading simulations with three cycles up to 15% strain in compression and tension at a constant strain rate of $0.01\;s^{-1}$. For the compression relaxation simulations in §3.3, we apply an almost instantaneous load of 15% compressive strain with a strain rate of $2.5\;s^{-1}$ and study the effect of different specimen radii r={4,6,8}mm. Finally, we use an inverse parameter identification algorithm (trust region reflective algorithm), as introduced in [38], to identify the best fitting material parameters to reproduce our experimental results introduced in §2.1 and §3.4.

## 3. Results

### 3.1. The volumetric stress contribution

**Figure 3.**
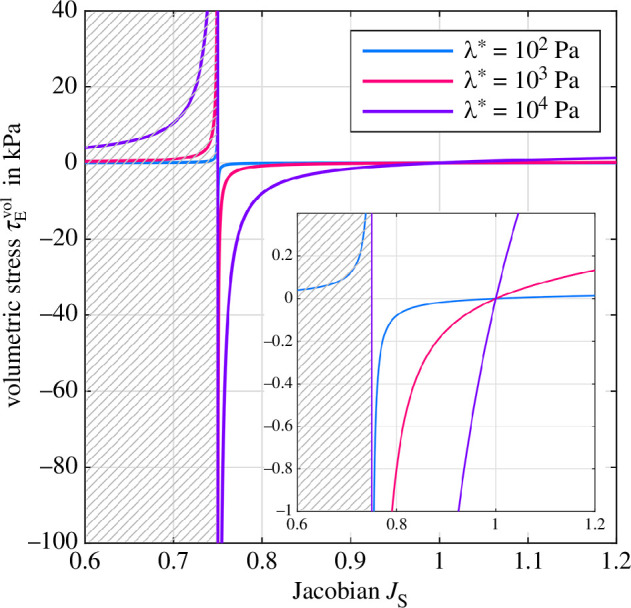
The effect of $\lambda^{*}=\{10^{2},10^{3},10^{4}\}\;\mathrm{Pa}$ on the volumetric tissue response depending on the Jacobian of the solid deformation gradient $J_{S}$ for an initial solid volume fraction of $n_{0S}^{S}=0.75$. The gray pattern indicates the non-admissible domain for $J_{S}<n_{0S}^{S}$. Insert: Magnification for $-1<{𝝉}_{E}^{\mathrm{vol}}<0.4\;\mathrm{kPa}$.

Figure 3 shows the volumetric stress response as a function of the Jacobian of the solid deformation gradient $J_{S}$ for an initial solid volume fraction of $n_{0S}^{S}=0.75$ and three different values of the first Lamé parameter $\lambda^{*}=\{10^{2},10^{3},10^{4}\}\;\mathrm{Pa}$. The additional term ${𝝉}_{E}^{\mathrm{vol}}$ in equation (2.5) primarily ensures incompressibility of the solid component once the compaction point is reached, i.e. ${𝝉}_{E}^{\mathrm{vol}}⟶-\infty$ when all fluid has left the biphasic material and $J_{S}⟶n_{0S}^{S}$. Therefore, the domain $J_{S}<n_{0S}^{S}$ is non-admissible, indicated by the grey pattern. But, depending on the choice of $\lambda^{*}$, ${𝝉}_{E}^{\mathrm{vol}}$ may add a significant volumetric constraint to the whole biphasic material over the entire deformation range—not only close to the compaction point. We clearly observe this effect in the inlet of figure 3, which shows the volumetric stress response in a range that is relevant for brain tissue with a stiffness of ≈1kPa. Here, high values of $\lambda^{*}$ already lead to a strong volumetric stress response for small volumetric changes of the biphasic material that in return constrains the actual fluid flow through the material. In other words, poroelastic effects are suppressed without changing the permeability of the material. As the permeability should control the fluid flow within a porous medium rather than the volumetric stress contribution, our analyses highlight that the choice of $\lambda^{*}$ should be made with caution and under consideration of the expected overall material response.

### 3.2. Poroelastic effects during cyclic loading

**Figure 4.**
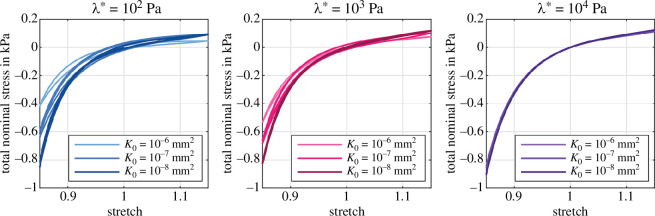
Poroelastic cyclic loading with Ogden parameters $\mu_{\infty }=-43.8\;\mathrm{Pa}$ and $\alpha_{\infty }=-12.76$ [38]. Effect of $\lambda^{*}=\{10^{2},10^{3},10^{4}\}\;\mathrm{Pa}$ on the overall tissue response for three different intrinsic permeabilities $K_{0}=\{10^{-6},10^{-7},10^{-8}\}\;{\mathrm{mm}}^{2}$.

Figure 4 shows the influence of the first Lamé parameter $\lambda^{*}$ on the poroelastic material behaviour for three different intrinsic permeabilities $K_{0}$ during cyclic loading. By way of example, we chose the equilibrium hyperelastic model parameters according to [38], who provided the Ogden parameters $\mu_{\infty }$ and $\alpha_{\infty }$ for the visual cortex based on an inverse parameter identification. We observe that with decreasing $\lambda^{*}$, the influence of the permeability on the overall material response increases. Specifically, for $\lambda^{*}=10^{2}\;\mathrm{Pa}$, the total nominal stress in compression increases significantly with decreasing permeability. On the contrary, for $\lambda^{*}=10^{4}\;\mathrm{Pa}$, the stress–stretch curves almost coincide and indicate that the permeability, and thus the porous material properties, do not affect the biphasic material response. We further recognize an increasing hysteresis with decreasing $\lambda^{*}$. The hysteresis originates from the porous energy dissipation and reaches a maximum for $\lambda^{*}=10^{2}\;\mathrm{Pa}$ and $K_{0}=10^{-7}\;{\mathrm{mm}}^{2}$ and decreases again for lower permeabilities [43].

**Figure 5.**
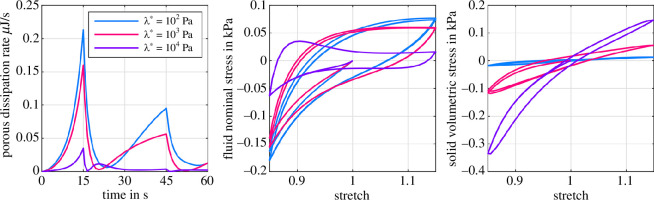
Poroelastic cyclic loading with Ogden parameters $\mu_{\infty }=-43.8\;\mathrm{Pa}$ and $\alpha_{\infty }=-12.76$ [38]. Effect of $\lambda^{*}=\{10^{2},10^{3},10^{4}\}\;\mathrm{Pa}$ on the porous dissipation rate $D_{p}$ of the first loading cycle (left), the fluid nominal stress $pJ_{S}1$ (middle) and the solid volumetric stress ${𝝉}_{E}^{\mathrm{vol}}$ (right) for an intrinsic permeability of $K_{0}=10^{-7}\;{\mathrm{mm}}^{2}$.

Figure 5 (left) highlights the effect of $\lambda^{*}$ on the porous dissipation rate, i.e. the hysteresis caused by porous effects, for the first compression–tension cycle (subsequent cycles display analogous responses). The porous dissipation rate increases for decreasing $\lambda^{*}$. In addition, the curve for $\lambda^{*}=10^{4}\;\mathrm{Pa}$ shows a significantly different behaviour. Here, the porous dissipation rate rapidly decreases to zero after the change of loading direction at 15s, followed by an intermediate maximum at ≈20s and another decrease in dissipation. Instead, for lower $\lambda^{*}$, we observe a delayed decrease in dissipation after the change of loading direction at 15s, followed by a monotonous increase until the next change of loading direction at 45s—without an intermediate maximum. Figure 5 (middle) shows that $\lambda^{*}$ not only affects the total material response but also the individual contribution of the fluid constituent. In particular, the fluid stress contribution increases with decreasing volumetric constraints, i.e. lower $\lambda^{*}$, accompanied by a larger hysteresis. Again, similar to the porous dissipation, the fluid stress response is entirely different for $\lambda^{*}=10^{4}\;\mathrm{Pa}$. Here, the maximum fluid tensile stress does not coincide with the maximum tensile stretch. Instead, we observe a maximum shortly after the change of loading direction, i.e. shortly after the maximum compressive strain has been reached.

Figure 5 (right) depicts the solid volumetric stress contribution induced by equation (2.10). As we can already expect from figure 3, the volumetric stress contribution almost vanishes for low values of $\lambda^{*}$. For high $\lambda^{*}$, it contributes up to one-third of the total biphasic material response, including a noticeable compression–tension asymmetry (compare with figure 4, right).

**Figure 6.**
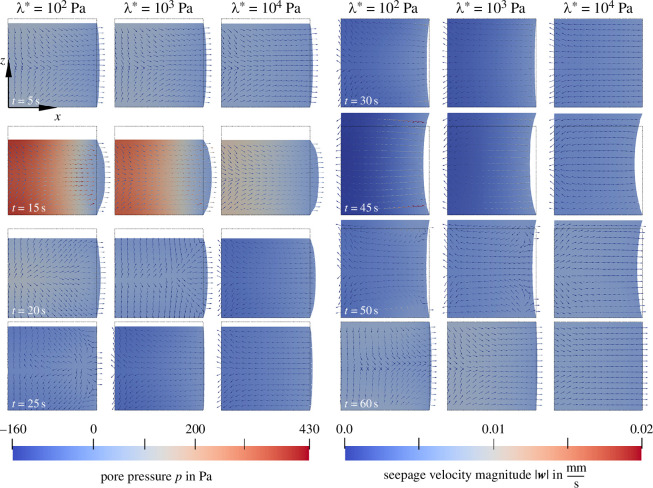
Poroelastic cyclic loading with Ogden parameters $\mu_{\infty }=-43.8\;\mathrm{Pa}$ and $\alpha_{\infty }=-12.76$ [38]. Effect of $\lambda^{*}=\{10^{2},10^{3},10^{4}\}\;\mathrm{Pa}$ on the pore pressure distribution and the seepage velocity 𝒘 (indicated by arrows) for an intrinsic permeability of $K_{0}=10^{-7}\;{\mathrm{mm}}^{2}$. The black dashed line indicates the undeformed configuration.

Figure 6 shows the pore pressure distribution and the seepage velocity in the x–z-plane of our cylindrical specimen at various time steps during the first loading cycle for different values of $\lambda^{*}=\{10^{2},10^{3},10^{4}\}\;\mathrm{Pa}$ and an intrinsic permeability of $K_{0}=10^{-7}\;{\mathrm{mm}}^{2}$. At t=15s, we reach the maximum compression at 15% strain. The pore pressure increases with decreasing $\lambda^{*}$, which indicates that the fluid takes a larger part of the load (compare figure 5, middle). The spatial distribution of the pore pressure is quite similar for different $\lambda^{*}$, but, due to the increasing pressure gradient, the seepage velocities increase for lower $\lambda^{*}$ (see red coloured arrows). After the change of loading direction, at t=20s, the fluid immediately flows back into the sample for high $\lambda^{*}$, while for low $\lambda^{*}$, even at t=25s, the fluid flow is not fully reversed. At t=30s, after half of the loading cycle, the specimen reaches its initial height. For high $\lambda^{*}$, the specimen volume is back to its initial volume, while the visible contraction for low $\lambda^{*}$ indicates a reduced specimen volume. Under 15% tensile strain at t=45s, we observe again higher pressure gradients and seepage velocities for lower $\lambda^{*}$. Similar to the situation after the first change of loading direction, the fluid flow into the sample continues for low $\lambda^{*}$ until the present pressure gradient decreases, while the flow reverses almost immediately for high $\lambda^{*}$. The first loading cycle is over after t=60s and the specimen shows a slightly increased volume for low $\lambda^{*}$.

### 3.3. Poroelastic effects during compression relaxation

**Figure 7.**
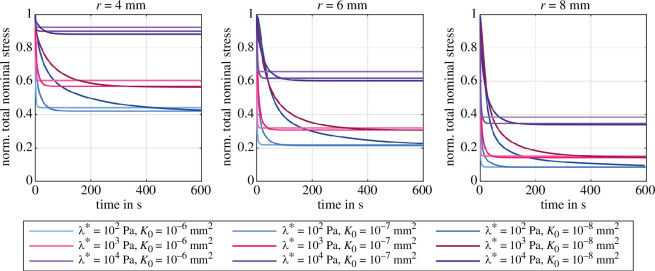
Poroelastic stress relaxation in compression with Ogden parameters $\mu_{\infty }=-43.8\;\mathrm{Pa}$ and $\alpha_{\infty }=-12.76$ [38]. Effect of $\lambda^{*}=\{10^{2},10^{3},10^{4}\}\;\mathrm{Pa}$ on the normalized total stress for three different intrinsic permeabilities $K_{0}=\{10^{-6},10^{-7},10^{-8}\}\;{\mathrm{mm}}^{2}$ and different specimen radii r={4,6,8}mm.

We choose a high loading rate of $2.5\;s^{-1}$ to approximately represent instantaneous loading and avoid that relaxation effects occur during loading. This way, we can further assume that the biphasic material acts almost as an incompressible elastic material during loading—independent of the choice of the first Lamé parameter $\lambda^{*}$. Figure 7 shows the simulated total nominal stress normalized by the peak loading stress during stress relaxation in compression for three different specimen radii r={4,6,8}mm, three different permeabilities $K_{0}=\{10^{-6},10^{-7},10^{-8}\}\;{\mathrm{mm}}^{2}$ and three different values of the first Lamé parameter $\lambda^{*}=\{10^{2},10^{3},10^{4}\}\;\mathrm{Pa}$. It highlights the size-dependency of nonlinear poroelasticity during stress relaxation with different specimen dimensions. The absolute amount of stress relaxation increases significantly with increasing specimen radius: from 10% to 60% for $\lambda^{*}=10^{4}\;\mathrm{Pa}$ and from 60% to 90% for $\lambda^{*}=10^{2}\;\mathrm{Pa}$. A larger specimen radius induces larger volumetric deformations, i.e. more fluid flow, more porous dissipation and larger stress relaxation. Increasing the first Lamé parameter $\lambda^{*}$ decreases the amount of stress relaxation, e.g. from 60% to 10% for r=4mm. This observation confirms our findings from §3.2 that increasing volumetric constraints diminish the porous effects. The permeability $K_{0}$ affects only the relaxation time, but not the total amount of relaxation. In most cases, we observe slightly less relaxation for the highest permeability $K_{0}=10^{-6}\;{\mathrm{mm}}^{2}$, which may indicate that still some relaxation has already happened during the loading phase.

**Figure 8.**
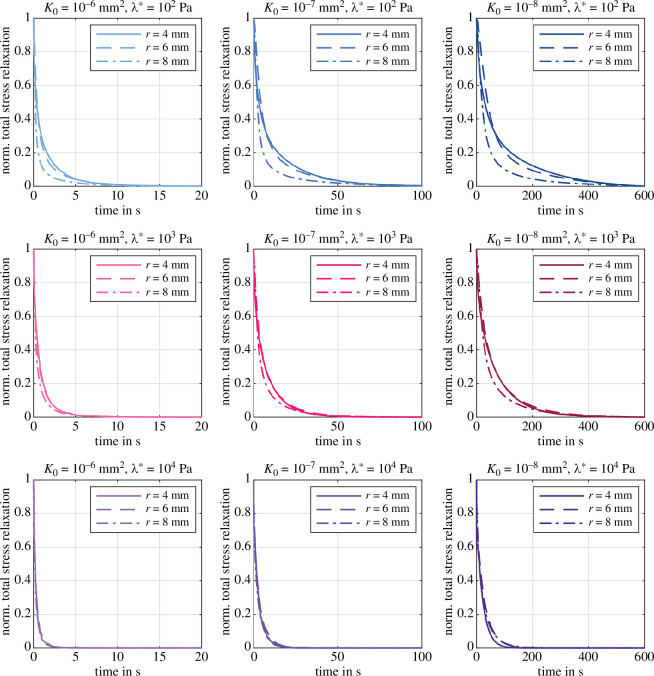
Poroelastic relaxation with Ogden parameters $\mu_{\infty }=-43.8\;\mathrm{Pa}$ and $\alpha_{\infty }=-12.76$ [38]. Effect of $\lambda^{*}=\{10^{2},10^{3},10^{4}\}\;\mathrm{Pa}$ on the normalized stress relaxation subtracted by the equilibrium stress for three different intrinsic permeabilities $K_{0}=\{10^{-6},10^{-7},10^{-8}\}\;{\mathrm{mm}}^{2}$ and different specimen radii r={4,6,8}mm.

Figure 8 shows the poroelastic relaxation behaviour for various combinations of the first Lamé parameter $\lambda^{*}$ and intrinsic permeabilities $K_{0}$ for three different specimen radii r={4,6,8}mm. The plots are normalized by the peak stress and subtracted by the equilibrium (relaxed) stress level after 600s holding time to solely focus on the relaxation behaviour.

First, the relaxation time decreases significantly with increasing $\lambda^{*}$. Large values for $\lambda^{*}$ impose large volumetric constraints on the biphasic material (§3.1), thus the admissible amount of volume change, and consequently the amount of induced fluid flow, decrease such that the material relaxes quicker. Second, the relaxation time increases considerably with decreasing permeability. This is an expected result, as the permeability controls the resistance of the fluid flow through the solid matrix. For constant $\lambda^{*}$, the applied displacement load induces the same amount of fluid flow, but decreasing permeability reduces the volume-weighted seepage velocity 𝒘 (see equation 2.11). Consequently, the relaxation process takes longer. Third, changes in specimen size seem to barely affect the time it takes to reach equilibrium, but changes the shape of the relaxation curve.

**Figure 9.**
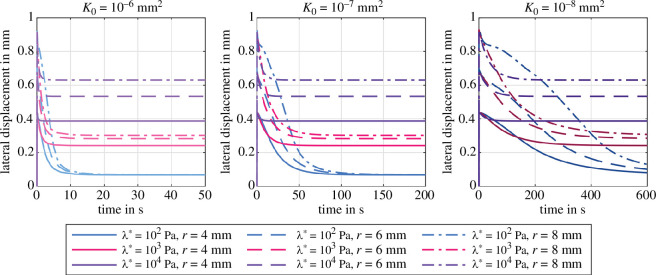
Poroelastic stress relaxation in compression with Ogden parameters $\mu_{\infty }=-43.8\;\mathrm{Pa}$ and $\alpha_{\infty }=-12.76$ [38]. Effect of $\lambda^{*}=\{10^{2},10^{3},10^{4}\}\;\mathrm{Pa}$ on the lateral displacement in x-direction for different intrinsic permeabilities $K_{0}=\{10^{-6},10^{-7},10^{-8}\}\;{\mathrm{mm}}^{2}$ and different specimen radii r={4,6,8}mm.

**Figure 10.**
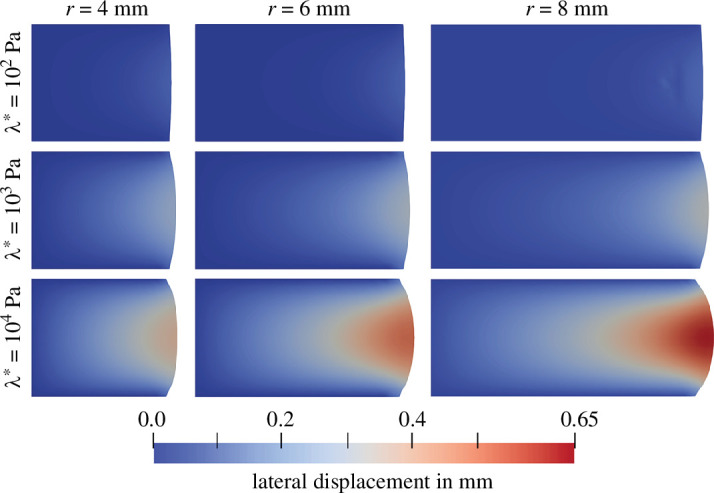
Deformation state after 600s of poroelastic stress relaxation in compression with Ogden parameters $\mu_{\infty }=-43.8\;\mathrm{Pa}$ and $\alpha_{\infty }=-12.76$ [38]. Effect of $\lambda^{*}=\{10^{2},10^{3},10^{4}\}\;\mathrm{Pa}$ on the lateral displacement for an intrinsic permeability $K_{0}=10^{-6}\;{\mathrm{mm}}^{2}$ and different specimen radii r={4,6,8}mm.

For low $\lambda^{*}$, increasing the radius leads to a quicker initial relaxation followed by a slower relaxation process. The size effect decreases with increasing $\lambda^{*}$ and even starts to show the opposite trend for $\lambda^{*}=10^{4}\;\mathrm{Pa}$ and the lowest permeability $K_{0}=10^{-8}\;{\mathrm{mm}}^{2}$. This behaviour could be related to the nonlinearity and deformation-dependency of our porous formulation. On the one hand, for high $\lambda^{*}$, the volumetric constraints lead to a rather homogeneous volumetric deformation of the specimen and thus a spatially homogeneous permeability throughout the specimen. Still, an increased specimen radius imposes larger volumetric changes and increases the pore pressure gradient from the specimen centre to its lateral surfaces. Following equation (2.11), the seepage velocity increases with the pressure gradient and compensates the increase in specimen size, such that the size-dependency of the relaxation time is less pronounced. On the other hand, for low $\lambda^{*}$, substantial volume changes occur and lead to an inhomogeneous permeability distribution in the specimen. In addition, increasing the radius leads to an increased initial lateral displacement (figures 9 and 10), increased solid strains, and increased solid stresses at the lateral surfaces. Therefore, the solid exerts a high pressure load on the fluid, which leads to an initially faster relaxation compared to smaller radii. Recall that this is not the case for high $\lambda^{*}$, since here a substantial part of this ‘additional’ load is taken by the volumetric stresses of the solid itself.

Figure 9 shows the lateral displacement, i.e. the ‘bulging out at half of the specimen height, where it reaches its maximum. Again, we study the influence of three permeabilities $K_{0}=\{10^{-6},10^{-7},10^{-8}\}\;{\mathrm{mm}}^{2}$, three Lamé parameters $\lambda^{*}=\{10^{2},10^{3},10^{4}\}\;\mathrm{Pa}$ and three specimen radii r={4,6,8}mm on the deformation and relaxation behaviour of the biphasic material. With decreasing permeability, the material deforms more slowly, but eventually reaches the same final deformation state. The initial maximum lateral displacement directly after loading remains almost unaffected by the permeability due to the high loading rate, i.e. volumetric changes during loading are negligible. In contrast, the first Lamé parameter $\lambda^{*}$ does not only affect the time required to reach the final deformation (compare figure 8), but also the final deformation state itself.

Figure 10 visualizes the final deformation state after 600s of holding time for a permeability of $K_{0}=10^{-6}\;{\mathrm{mm}}^{2}$, and different combinations of Lamé parameters and specimen radii. The lateral displacement in the fully relaxed state increases significantly with increasing $\lambda^{*}$, as it controls the fluid flow and volume change of the biphasic material. In fact, the lateral displacement almost vanishes for $\lambda^{*}=10^{2}\;\mathrm{Pa}$—a behaviour we would typically expect from a material with a Poisson’s ratio close to zero— while, depending on the radius, up to 0.65mm lateral displacement remain for $\lambda^{*}=10^{4}\;\mathrm{Pa}$. In conjunction with high $\lambda^{*}$, larger specimen radii naturally increase the final lateral displacement as they induce larger volumetric changes, see figure 9. The radius itself does increase the initial lateral displacement.

**Figure 11.**
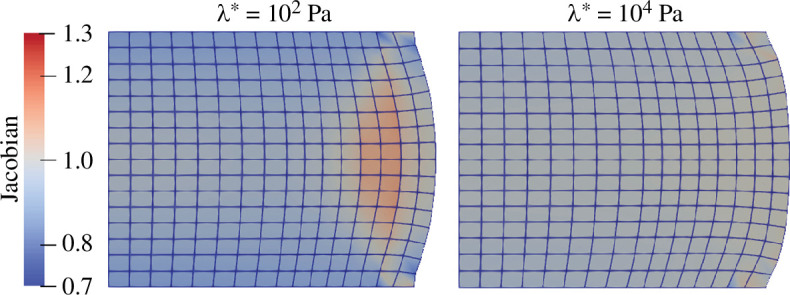
Deformation state after 60s of poroelastic stress relaxation in compression with Ogden parameters $\mu_{\infty }=-43.8\;\mathrm{Pa}$ and $\alpha_{\infty }=-12.76$ [38]. Effect of $\lambda^{*}=\{10^{2},10^{4}\}\;\mathrm{Pa}$ on the local volumetric changes for an intrinsic permeability $K_{0}=10^{-8}\;{\mathrm{mm}}^{2}$ and a specimen radius of r=4mm.

Interestingly, for low permeabilities and low $\lambda^{*}$, the temporal progression of the lateral retraction changes, maintaining a larger lateral displacement in the beginning of the relaxation followed by an accelerated decline, see figure 9 right. This observation is counterintuitive, as we would expect a behaviour similar to the stress relaxation in figure 8. Recalling the solid volumetric stress contribution from the extension function in figure 3 of our poroelastic model, low values of $\lambda^{*}$ facilitate local volumetric changes. As depicted in figure 11, left, this especially includes the possibility of volumetric expansion, i.e. local accumulation of fluid. Driven by the large pore pressure in the specimen centre, fluid quickly leaves the inner part of the specimen and then accumulates close to the boundary. In this area, where $J_{S}>1$, the resistance of the solid matrix against volumetric expansion becomes smaller than the fluid flow resistance due to the permeability. This way, the fluid contribution to the total stress reduces while maintaining a large lateral displacement. In contrast, high values of $\lambda^{*}$ impede volumetric changes leading to less fluid flow and a more homogeneous distribution of volumetric deformations, see figure 11 (right).

### 3.4. Region-dependent inverse parameter identification

To see how our insights obtained from the parameter studies in the previous sections transfer to real experimental data introduced in §2.1, we apply an inverse parameter identification scheme to identify the best fitting material parameters for two regions of the human brain: the grey matter visual cortex and the white matter corona radiata. For details regarding the inverse parameter identification algorithm, we refer to [38].

**Figure 12.**
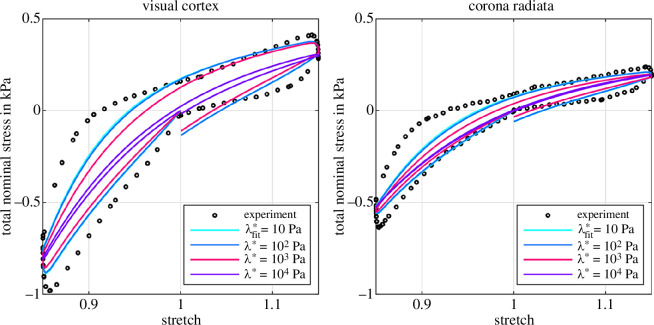
Inverse poroelastic parameter identification for the visual cortex (left) and the corona radiata (right). Parameters ($\mu_{\infty }$, $\alpha_{\infty }$, $K_{0}$) are identified for four different first Lamé parameters $\lambda^{*}=\{\lambda_{\mathrm{fit}}^{*},10^{2},10^{3},10^{4}\}\;\mathrm{Pa}$ (see tables 1 and 2). $\lambda_{\mathrm{fit}}^{*}=10\;\mathrm{Pa}$ shows the result with $\lambda^{*}$ as an additional parameter for the optimization algorithm.

Figure 12 shows the results of the inverse parameter identification for cyclic compression–tension with a purely poroelastic material model for the visual cortex and the corona radiata. For each brain region, we perform three fits with fixed values for $\lambda^{*}=\{10^{2},10^{3},10^{4}\}\;\mathrm{Pa}$ to determine three unknown material parameters: the Ogden shear modulus $\mu_{\infty }$, the Ogden nonlinearity parameter $\alpha_{\infty }$, and the isotropic initial intrinsic permeability $K_{0}$. In addition, we run one fit with $\lambda_{\mathrm{fit}}^{*}$ as a fourth parameter that is identified by the optimization algorithm. In accordance with our observations in §3.2, the highest values for $\lambda^{*}=10^{4}\;\mathrm{Pa}$ lead to the highest model prediction errors, indicated by a root mean square error (RMSE) of 165Pa for the visual cortex and 94Pa the corona radiata, respectively. For $\lambda^{*}=10^{4}\;\mathrm{Pa}$, the volumetric constraints on the biphasic material suppress the actual porous behaviour and prevent almost any hysteresis.

Decreasing $\lambda^{*}$ improves the quality of the fits significantly and our poroelastic model proves its ability to capture important mechanical characteristics of brain tissue, i.e. compression–tension asymmetry and—to some extent—hysteresis. The optimization algorithm identifies an even lower $\lambda_{\mathrm{fit}}^{*}=10\;\mathrm{Pa}$ as the best possible choice. We note that this is the lower limit of admissible values we set for $\lambda^{*}$ to avoid numerical problems. Nevertheless, the quality of the fit does not change noticeably—the curves for $\lambda_{\mathrm{fit}}^{*}=10\;\mathrm{Pa}$ and $\lambda^{*}=10^{2}\;\mathrm{Pa}$ almost coincide (see also RMSE in tables 1 and 2)—indicating that we approach the limits of the poroelastic formulation.

**Table 1.** Inverse poroelastic parameter identification for the visual cortex. Parameters ($\mu_{\infty }$, $\alpha_{\infty }$, $K_{0}$) are identified for four different first Lamé parameters $\lambda^{*}=\{10^{2},10^{3},10^{4}\}\;\mathrm{Pa}$. $\lambda_{\mathrm{fit}}^{*}=10\;\mathrm{Pa}$ shows the result with $\lambda^{*}$ as an additional parameter for the optimization algorithm.

visual cortex				
	$\mu_{\infty }\;(Pa)$	$\alpha_{\infty }\;(-)$	$K_{0}\;({mm}^{2})$	RMSE(Pa)
$\lambda_{fit}^{*}=10\;Pa$	−1180	−2.38	$1.27\times 10^{-7}$	84
$\lambda^{*}=10^{2}\;Pa$	−1120	−2.43	$1.21\times 10^{-7}$	88
$\lambda^{*}=10^{3}\;Pa$	−714	−3.00	$8.46\times 10^{-8}$	115
$\lambda^{*}=10^{4}\;Pa$	−347	−3.74	$2.50\times 10^{-8}$	165

**Table 2.** Inverse poroelastic parameter identification for the corona radiata. Parameters ($\mu_{\infty }$, $\alpha_{\infty }$, $K_{0}$) are identified for four different first Lamé parameters $\lambda^{*}=\{10^{2},10^{3},10^{4}\}\;\mathrm{Pa}$. $\lambda_{\mathrm{fit}}^{*}=10\;\mathrm{Pa}$ shows the result with $\lambda^{*}$ as an additional parameter for the optimization algorithm.

corona radiata				
	$\mu_{\infty }\;(Pa)$	$\alpha_{\infty }\;(-)$	$K_{0}\;({mm}^{2})$	RMSE(Pa)
$\lambda_{fit}^{*}=10\;Pa$	−498	−3.52	$1.89\times 10^{-7}$	57
$\lambda^{*}=10^{2}\;Pa$	−457	-3.67	$1.73\times 10^{-7}$	60
$\lambda^{*}=10^{3}\;Pa$	−256	−4.93	$9.95\times 10^{-8}$	76
$\lambda^{*}=10^{4}\;Pa$	−266	-3.38	$4.01\times 10^{-8}$	94

Tables 1 and 2 summarize the identified material parameters for cyclic compression–tension and the purely poroelastic case. Both the Ogden shear modulus $\mu_{\infty }$ and the initial intrinsic permeability $K_{0}$ decrease with increasing first Lamé parameter, while the nonlinearity slightly increases. This highlights that $\lambda^{*}$ influences the solid and the fluid behaviour within the biphasic material. The permeability is slightly lower for the visual cortex than for the corona radiata but in the same order of magnitude.

**Figure 13.**
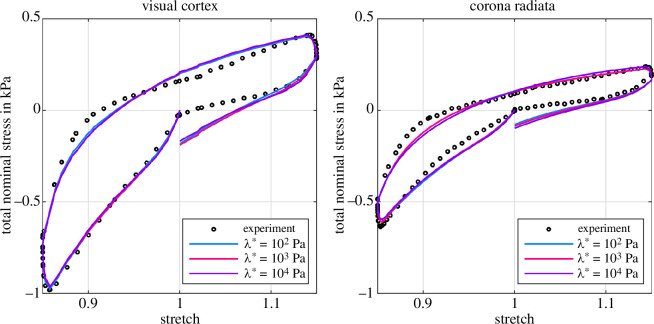
Inverse poro-viscoelastic parameter identification for the visual cortex and the corona radiata. Parameters ($\mu_{\infty }$, $\alpha_{\infty }$, $\mu_{1}$, $\alpha_{1}$, η, $K_{0}$) are identified for three different first Lamé parameters $\lambda^{*}=\{10^{2},10^{3},10^{4}\}\;\mathrm{Pa}$.

As the purely poroelastic model is not able to capture the highly hysteretic response of brain tissue, we add a viscoelastic element to the solid part of our model (§2.4). Figure 13 shows the results of our inverse parameter identification for cyclic compression–tension for the poro-viscoelastic case. Again, we identify the best fitting material parameters for three different first Lamé parameters $\lambda^{*}=\{10^{2},10^{3},10^{4}\}\;\mathrm{Pa}$. The optimization algorithm determines the equilibrium and non-equilibrium Ogden parameters $\mu_{\infty },\alpha_{\infty },\mu_{1},\alpha_{1}$, the solid viscosity η, and the initial intrinsic permeability $K_{0}$. For both brain regions, the poro-viscoelastic model improves the quality of the fits significantly compared to the purely poroelastic model. Interestingly, the fits almost coincide for different values of the first Lamé parameter $\lambda^{*}$, indicating that viscoelastic effects dominate the fitted material response.

Tables 3 and 4 display the best fitting poro-viscoelastic material parameters for cyclic compression-tension. The equilibrium Ogden parameters for the visual cortex are similar to those of the purely poroelastic model. The corresponding non-equilibrium parameters increase the material nonlinearity and indicate a significant strain-rate dependency of the tissue. The solid viscosity decreases with increasing $\lambda^{*}$. The equilibrium Ogden parameters for the corona radiata differ slightly from those for the purely poroelastic model. For all $\lambda^{*}$, the equilibrium Ogden shear modulus $\mu_{\infty }$ decreases to similar values and the nonlinearity parameter $\alpha_{\infty }$ increases. The solid viscosity decreases with increasing $\lambda^{*}$. For both brain regions, the initial intrinsic permeability $K_{0}$ is similar to that of the poroelastic model for low $\lambda^{*}$, while it changes by several orders of magnitude for high $\lambda^{*}$. This further demonstrates that high values of $\lambda^{*}$ suppress porous effects, such that the permeability loses its influence on the biphasic material response and might be chosen almost arbitrarily.

**Table 3.** Inverse poro-viscoelastic parameter identification for the visual cortex. Parameters ($\mu_{\infty }$, $\alpha_{\infty }$, $\mu_{1}$, $\alpha_{1}$, η, $K_{0}$) are identified for three different first Lamé parameters $\lambda^{*}=\{10^{2},10^{3},10^{4}\}\;\mathrm{Pa}$.

visual cortex							
	$\mu_{\infty }\;(Pa)$	$\alpha_{\infty }\;(-)$	$\mu_{1}\;(Pa)$	$\alpha_{1}\;(-)$	η(Pa⋅s)	$K_{0}\;({mm}^{2})$	RMSE (Pa)
$\lambda^{*}=10^{2}\;Pa$	−1010	−2.78	-1170	−6.31	5380	$1.86\times 10^{-7}$	45
$\lambda^{*}=10^{3}\;Pa$	−683	−3.18	−791	-10.7	5230	$1.52\times 10^{-7}$	52
$\lambda^{*}=10^{4}\;Pa$	−404	-2.94	−1040	-8.85	3300	$2.28\times 10^{-10}$	49

**Table 4.** Inverse poro-viscoelastic parameter identification for the corona radiata. Parameters ($\mu_{\infty }$, $\alpha_{\infty }$, $\mu_{1}$, $\alpha_{1}$, η, $K_{0}$) are identified for three different first Lamé parameters $\lambda^{*}=\{10^{2},10^{3},10^{4}\}\;\mathrm{Pa}$.

corona radiata							
	$\mu_{\infty }\;(Pa)$	$\alpha_{\infty }\;(-)$	$\mu_{1}\;(Pa)$	$\alpha_{1}\;(-)$	η(Pa⋅s)	$K_{0}\;({mm}^{2})$	RMSE (Pa)
$\lambda^{*}=10^{2}\;Pa$	−173	-5.57	−810	-3.70	3220	$5.75\times 10^{-9}$	32
$\lambda^{*}=10^{3}\;Pa$	-149	−5.96	-546	−6.18	3120	$2.87\times 10^{-9}$	32
$\lambda^{*}=10^{4}\;Pa$	−181	-4.17	−2970	-1.46	2650	$7.57\times 10^{-4}$	38

**Figure 14.**
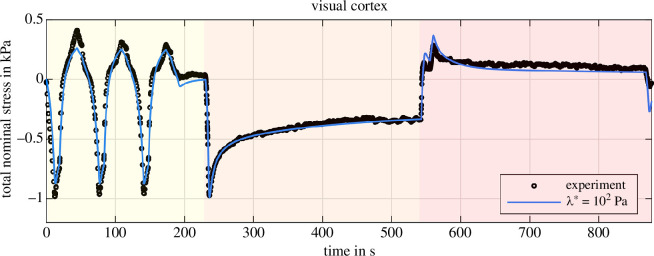
Inverse poro-viscoelastic parameter identification for the visual cortex. Three loading cycles (yellow), followed by compression (orange) and tension (red) relaxation. Parameters ($\mu_{\infty }$, $\alpha_{\infty }$, $\mu_{1}$, $\alpha_{1}$, η, $K_{0}$) are identified for $\lambda^{*}=10^{2}\;\mathrm{Pa}$.

**Figure 15.**
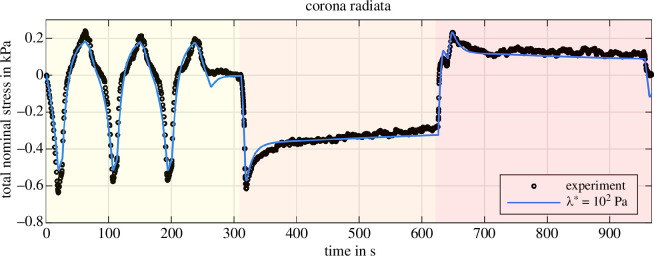
Inverse poro-viscoelastic parameter identification for the corona radiata. Three loading cycles (yellow), followed by compression (orange) and tension (red) relaxation. Parameters ($\mu_{\infty }$, $\alpha_{\infty }$, $\mu_{1}$, $\alpha_{1}$, η, $K_{0}$) are identified for $\lambda^{*}=10^{2}\;\mathrm{Pa}$.

So far, our results suggest that a single compression-tension cycle is insufficient to reliably quantify poro-viscoelastic material parameters. Therefore, we apply our inverse parameter identification scheme to larger experimental datasets. We fit the experimental response for three subsequent compression–tension loading cycles, followed by compression and tension relaxation. Thereby, we provide additional information on the conditioning behaviour and include two different displacement rates, i.e. $40\;\mu m\;s^{-1}$ (cyclic loading) and $100\;\mu m\;s^{-1}$ (relaxation test). Figures 14 and 15 show the fitted material response of the visual cortex and corona radiata, respectively, for $\lambda^{*}=10^{2}\;\mathrm{Pa}$. Table 5 shows the corresponding best fitting material parameters. Compared to the poro-viscoelastic parameters in table 3, the equilibrium and non-equilibrium shear moduli decrease significantly while the corresponding nonlinearity parameters increase for the visual cortex. The solid viscosity increases slightly and the permeability decreases by two orders of magnitude. For reduced permeability, the fluid takes longer to flow out of the sample, indicating that it controls the long-term material response. We observe similar but less pronounced trends for the corona radiata in comparison with table 4.

**Table 5.** Inverse poro-viscoelastic parameter identification for the visual cortex (VC) and the corona radiata (CR). Three loading cycles, followed by compression and tension relaxation. Parameters ($\mu_{\infty }$, $\alpha_{\infty }$, $\mu_{1}$, $\alpha_{1}$, η, $K_{0}$) are identified for $\lambda^{*}=10^{2}\;\mathrm{Pa}$.

region	$\lambda^{*}\;(Pa)$	$\mu_{\infty }\;(Pa)$	$\alpha_{\infty }\;(-)$	$\mu_{1}\;(Pa)$	$\alpha_{1}\;(-)$	η(Pa⋅s)	$K_{0}\;({mm}^{2})$	RMSE (Pa)
VC	$10^{2}$	-30.7	−11.9	-45.9	−14.4	6770	$4.18\times 10^{-9}$	62
CR	$10^{2}$	−81.0	-7.60	−494	-1.53	3860	$5.16\times 10^{-9}$	46

The overall quality of the fit is very satisfactory for such a highly complex material as human brain tissue. We almost perfectly capture the material behaviour under compression and during the relaxation tests for the visual cortex (figure 14). But, we underestimate the stress under tension—especially in the first loading cycle—and cannot reproduce the conditioning behaviour under tension. An almost perfect fit is observed for the corona radiata under tensile loading and the relaxation behaviour is captured reasonably well, while we slightly underestimate the material response under compression (figure 15). All in all, while our poro-viscoelastic model captures the cyclic loading and relaxation behaviour reasonably well, for the identified set of parameters, the model cannot reproduce the conditioning behaviour.

## 4. Discussion

In this study, we have investigated the physical meaning of poroelastic parameters and their interplay with viscous effects in the context of a poro-viscoelastic model following the Theory of Porous Media. Based on our analyses, we have identified a set of model parameters for two different regions of the human brain, visual cortex (gray matter) and corona radiata (white matter), through an inverse approach using the finite element method and experimental data from cyclic and stress relaxation loadings. The identified parameters hint towards differences between the individual contributions of viscous and porous effects in gray and white matter, which can also explain the large variation of corresponding measured properties in the literature that highly depend on the experimental setup and loading conditions. Finally, we have highlighted critical points for the reliable quantification of poro-viscoelastic material parameter sets in the future.

### 4.1. The role of the parameter $\lambda^{*}$

The first Lamé parameter $\lambda^{*}$ plays a crucial role in the model-predicted overall behaviour of biphasic human brain tissue. Decreasing $\lambda^{*}$ increases the sensitivity of the model to the permeability $K_{0}$, while large values of $\lambda^{*}$ constrain volumetric changes of the biphasic material and lead to diminishing porous effects, less deformation-dependency of the fluid response (see equation 2.11), less model nonlinearity and a more homogeneous material behaviour. From a physical perspective, the permeability should significantly affect the material resistance to compressive loading (e.g. when squeezing a sponge). The lower the permeability, the more fluid remains ‘trapped’ within the tissue, which generates higher hydrostatic (i.e. volumetric) stress, and hence, larger total nominal stresses. Our results show that high values of $\lambda^{*}$, around one order of magnitude higher than the classical shear modulus, constrain fluid flow within the tissue and thus prevent physically reasonable behaviour, including almost any porous dissipation during cyclic loading (compare figure 4, right). Reducing $\lambda^{*}$ allows larger volumetric deformations and fluid flow. This introduces a significant time dependence of the poroelastic material response (see deformation states in figure 6) controlled by the permeability. Therefore, the choice of $\lambda^{*}$ should be made with caution and under consideration of the expected overall material stiffness and compressibility.

In comparison to other material model parameters in the literature, the first Lamé parameter has a qualitatively similar effect to the Poisson’s ratio ν. Therefore, we can relate our findings to [44] mentioning less volume change, less interstitial fluid movement and less fluid flow-dependent viscoelasticity in articular cartilage for ν→0.5, i.e. high $\lambda^{*}$. In addition, [45], observe a decreasing fluid contribution with increasing Poisson’s ratio during rat brain indentation. In contrast [31], set a rather high Poisson’s ratio, ν=0.49.

Be aware that we assume the elastic stress tensor ${𝝉}_{E}^{\mathrm{eq}}$ has both volumetric and isochoric contributions (see equation 2.6), which implies that the equilibrium shear modulus $\mu_{\infty }$ may influence part of the volumetric response of the tissue. However, our simulations to date suggest that the volumetric stress response is primarily driven by ${𝝉}_{E}^{\mathrm{vol}}$ and $pJ_{S}1$, hence we focus on the parameter $\lambda^{*}$. A deeper understanding of these potential, hidden interactions could affect how we interpret the influence of the parameters.

Note that the choice of the first Lamé parameter $\lambda^{*}$ might become less crucial under different loading conditions. For example, the parameter sensitivity study performed by [23] found that $\lambda^{*}$ was not determinant. However, their model considers a triphasic material (solid skeleton, interstitial fluid and blood) with mass-production terms added to the mass balance equation that represent a series of cellular mechanisms. Therefore, the parameters controlling these added terms have a larger influence on the results. Another case is a perfusion setup, where primarily an externally applied fluid pressure drives the fluid flow through the tissue and deformation-driven fluid flow becomes secondary, i.e. no significant material deformation occurs. But, this would require a comparably stiff solid matrix in combination with rather high permeabilities, which is certainly not the case for the ultrasoft human brain tissue.

### 4.2. Poroelastic effects during stress relaxation experiments

Due to the high loading rate, the biphasic poroelastic material responds like an incompressible elastic material during the loading phase of the relaxation simulations. This results in almost identical stress-strain states before the actual relaxation process starts, independent of the choice of the first Lamé parameter $\lambda^{*}$ and the initial intrinsic permeability $K_{0}$. In this initial state, the resistance of the biphasic material to volumetric changes is almost exclusively determined by the fluid constituent, which bears the pressure load. During the relaxation, two processes occur simultaneously. Due to the pressure exerted by the solid on the fluid, fluid starts flowing out of the specimen with a seepage velocity regulated by the intrinsic permeability. At the same time, as fluid content is reduced in the biphasic material, the pressure load on the fluid is gradually transferred to the solid volumetric stresses. This process is governed by the extension function (see equation 2.10), and accelerates with increasing $\lambda^{*}$. The larger $\lambda^{*}$, the less volumetric changes are allowed, and the earlier the pressure load on the fluid transfers to the solid volumetric stresses.

Increasing $\lambda^{*}$ leads to higher equilibrium stresses and less porous dissipation, i.e. relaxation, while the permeability controls the shape of the curve, i.e. the relaxation times (compare figure 7). Increasing the specimen radius while keeping the specimen height constant induces larger volumetric deformations and the effects of porous dissipation and corresponding relaxation increase in importance. In contrast to other studies [46], the time until equilibrium is reached does not appear to increase with increasing radius. In fact, for high values of $\lambda^{*}$, the relaxation behaviour is not affected by the radius, while the shape of the relaxation curve changes with the radius for lower values of $\lambda^{*}$ (figure 8). This could be related to the high degree of nonlinearity within our model, as [47] report on much higher stress relaxation in rat brain tissue than predicted by a linear biphasic theory. Larger deformation leads to higher solid stresses and higher pore pressure. Subsequently, larger pressure gradients induce higher seepage velocities that compensate for the larger distances the fluid has to move through in a larger specimen.

Importantly, the first Lamé parameter $\lambda^{*}$ not only affects the equilibrium stress during relaxation experiments, but also the temporal evolution of the specimen geometry (figure 9). For high values of $\lambda^{*}$, the ‘bulging out’ of the sample during compression (when the specimen is glued to the upper and lower specimen holder) remains, even after a holding time of 600 s, as $\lambda^{*}$ constrains volumetric changes due to fluid flow. For low values of $\lambda^{*}$, more fluid can flow out of the sample, so that the sample almost forms back to a cylindrical shape during the holding time. Note that these effects are also strongly coupled to the deformation-dependent permeability, which decreases with decreasing specimen volume.

Incorporating a precise camera setup to capture the specimen geometry and deformation during relaxation experiments can in the future help to choose an appropriate $\lambda^{*}$ value for poroelastic materials (see also [48]). But, due to the relatively long timescales involved, it remains to be seen whether effects like tissue degradation or swelling need to be taken into account to obtain reliable results.

### 4.3. Poro-viscoelastic material parameters for human brain tissue

While a purely poroelastic model is unable to capture the highly hysteretic response of brain tissue (compare figure 12), we show that a poro-viscoelastic material model can capture the response of human brain tissue during both cyclic loading and stress relaxation experiments in compression and tension (see figures 14 and 15). Two coupled processes control the time-dependent tissue response: viscous effects are responsible for the short-term relaxation and porous effects take over for the long-term relaxation behaviour. Similar observations have been previously reported for calve white matter [27] and articular cartilage [44].

As discussed in the previous subsections, high values of $\lambda^{*}$ suppress porous effects, such that the permeability loses its influence on the biphasic material response and might be chosen almost arbitrarily during an inverse parameter identification. Therefore, we purposely chose a low value for $\lambda^{*}$ to identify all remaining model parameters. We obtained an initial intrinsic permeability $K_{0}$ of $4.18\times 10^{-9}\;{mm}^{2}$ for the visual cortex and $5.16\times 10^{-9}\;{mm}^{2}$ for the corona radiata. Table 6 summarizes permeability measures of brain tissue that have been used previously and shows that our identified permeabilities are within a reasonable range. Overall, we observe slightly lower permeabilities for grey matter (visual cortex) than white matter (corona radiata). Differences in permeability alter the biphasic material response to different loading rates: for very small and intermediate loading rates, more fluid flows through white matter tissue and is squeezed out than in grey matter. As a result, white matter appears softer than grey matter under slow and intermediate loading conditions. For extremely fast loading in turn, white matter becomes stiffer as now trapped fluid contributes to the stiffness and the difference between grey and white matter becomes less pronounced or even inverted. For example, during magnetic resonance elastography at very high frequencies, corona radiata has been reported to be stiffer than the cortex [56,57].

**Table 6.** Values for brain tissue permeability used throughout the literature for different tissue types: white matter (WM), grey matter (GM) and mixed matter (MM). Note that experiments typically provide hydraulic permeability, which can be directly related to the intrinsic permeability used in our model. Units have been converted under the assumption of $\mu_{\mathrm{FR}}=10^{-3}\;\mathrm{Pa}\cdot s$ (effective shear viscosity of water at $20^{\circ }C$) and $\gamma_{FR}=10^{4}\;Nm^{-3}$ (specific weight of water).

study	tissue type	permeability $K_{0}$ in mm^2^
[27]	calve, WM	$4.08\times 10^{-9}$
[19]	human, WM	$2.4\times 10^{-11}$
Greiner *et al*. (present study)	human, WM	$5.16\times 10^{-9}$
Greiner *et al*. (present study)	human, GM	$4.18\times 10^{-9}$
[47]	rat, MM	$3.56\times 10^{-6}\;\ldots \;2.22\times 10^{-12}$
[31]	bovine	$1.57\times 10^{-9}$
[49]	ovine, WM	$0.7\ldots 2.0\times 10^{-10}$
[50]	human, WM	$1.6\times 10^{-8}$
[50]	human, GM	$1.6\times 10^{-10}$
[30]	human	$2.19\times 10^{-9}$
[51]	human	$1.4\times 10^{-8}$
[52]	rat, MM	$6.4\times 10^{-12}$
[53]	sheep, WM	$0.43\ldots 1.71\times 10^{-12}$
[45]	rat, 3 regions	$1.2\ldots 5.5\times 10^{-10}$
[54]	ovine, WM	$1.3\times 10^{-8}\;\ldots \;2.0\times 10^{-9}$
[55]	human, WM	$6.5\times 10^{-9}$

Equally as important as the porous effect is the second time-dependent process controlled by the solid’s viscosity. Our fits indicate a 75% higher viscosity for gray matter than white matter, i.e. faster relaxation for the corona radiata. Again, this could explain why white matter appears softer for slow loading rates and stiffer than gray matter for high loading rates. In addition, not only the loading rate but also the loading magnitude can impede the comparability between different experimental setups. For example, for the corona radiata, our fit suggests a distinctively lower nonlinearity of the non-equilibrium part in combination with a higher non-equilibrium shear modulus. This indicates a stiffer response in the small-strain regime compared to the visual cortex.

### 4.4. Limitations and future perspectives

We note that our inversely identified parameters are not yet unique. For example, the porous and viscous timescales could switch such that the porous effects dominate the short-term relaxation and the viscous effects control the long-term relaxation behaviour. This would still allow us to satisfactorily fit the material response, but the underlying physical material behaviour would change completely, i.e. large and rapid volumetric changes would occur. The non-uniqueness resides on the model complexity and shows that additional experimental data are required to reliably calibrate all poro-viscoelastic material parameters. The strong coupling between porous, viscous and volumetric effects in conjunction with the ultrasoft and fragile nature of human brain tissue poses immense challenges to future experimental setups. Specific perfusion experiments that precisely trigger and measure fluid flow through the brain on the tissue scale, combined with tracking local deformation states, appear most promising to identify the first Lamé parameter $\lambda^{*}$ and the permeability $K_{0}$. Thereby, the aim should be to keep the overall deformation as small as possible—while maintaining a measurable and stable fluid flow—and to reduce the loading rate such that viscous effects can be neglected. With these parameters at hand, complementary large-strain cyclic loading and relaxation tests can provide the missing viscous material properties and a digital image correlation system could serve as a validation for the local deformation states. Kainz *et al*. [58] showed that brain tissue-mimicking materials with very similar mechanical properties provide the great opportunity to design and calibrate such new experimental setups while adhering to ethical principles.

## Data Availability

The original code that was the basis for this work and updates related to this paper are available in the deal.ii code gallery: [59]. The updated code and experimental data used for the inverse parameter identification are available on Zenodo: [60].
